# Detecting gender bias in Arabic text through word embeddings

**DOI:** 10.1371/journal.pone.0319301

**Published:** 2025-03-31

**Authors:** Aya Mourad, Fatima K. Abu Salem, Shady Elbassuoni

**Affiliations:** Computer Science Department, American University of Beirut, Beirut, Lebanon; Khalifa University, UNITED ARAB EMIRATES

## Abstract

For generations, women have fought to achieve equal rights with those of men. Many historians and social scientists examined this uphill path with a focus on women’s rights and economic status in the West. Other parts of the world, such as the Middle East, remain understudied, with a noticeable shortage in gender-based statistics in the economic arena. According to the sociocognitive theory of critical discourse analysis, social behaviors and norms are reflected by language discourses, which motivates the present study, where we examine gender-based biases in various occupations, as reflected through various textual corpora. Several works in literature have shown that word embedding models can learn biases from the textual data they are trained on, which can propagate societal prejudices that have been implicitly embedded in such text. In our study, we adapt WEAT and Direct Bias quantification tests for Arabic, to examine gender bias with respect to a wide set of occupations as reflected in various Arabic text datasets. These datasets include two Lebanese news archives, Arabic Wikipedia, and electronic newspapers in UAE, Egypt, and Morocco, thus providing different outlooks into female and male engagements in various professions. Our WEAT tests across all datasets indicate that words related to careers, science, and intellectual pursuits are linked to men. In contrast, words related to family and art are associated with women across all datasets. The Direct Bias analysis shows a consistent female gender bias towards professions such as nurse, house cleaner, maid, secretary, and dancer. As the Moroccan News Articles Dataset (MNAD) showed, females were also associated with additional occupations such as researcher, doctor, and professor. Considering that the Arab world remains short on census data exploring gender-based disparities across various professions, our work provides evidence that such stereotypes persist till this day.

## Introduction

The Arab region has the world’s lowest rate of female labor force participation - 18.4% compared to the global average of 48% [20]. Several factors have been alleged in various literature as contributing to the gender discrepancies that persist in the Arab world today, including the employment sector. Such factors include patriarchal cultures, the legacy of colonialism, and rigid religious interpretations, all of which point to societal ideologies and biases [2]. In this manuscript, we explore indicators of gender-driven biases with respect to occupations, as reflected in discourse in Arabic text. To enable large scale and automated critical discourse analysis, our focal point of inquiry will revolve around the biases reflected in word embeddings. Word embeddings have emerged as an effective tool in natural language processing, where they have been used in various tasks such as sentiment analysis, machine translation, document ranking, etc. Yet, despite their tremendous potential, many studies show that word embeddings comprehend the biases in the data on which they are trained, which encapsulates societal preconceptions and prejudices [3,5,29,33].

Most existing studies focus on quantifying, analyzing, and mitigating gender bias in English word embeddings and English text. Many methods have been developed to compute bias, including the Direct Bias method [3] and the WEAT test [5]. Boulbaski et al. [3] utilized a definitional set of gender word pairs to establish a gender subspace within word embeddings using Principal Component Analysis (PCA). They then identified the gender direction by extracting the component of the highest variance. The bias is calculated by projecting the vector of a neutral word into the gender direction. For example, “nurse" is biased as it tends to lean towards the female direction when projected on the gender direction. Clasikan et al. [5] proposed a statistical test named Word Embedding Association Test (WEAT) designed to quantify bias by enabling the measurement of the strength of association between a set of target words representing a particular group (e.g., gender, race) and a set of attribute words depicting specific stereotypes.

Some other approaches to identify gender bias in word embeddings also exist. Dev and Phillips [7] introduced the Embedding Coherence Test (ECT) to measure whether two sets of target terms exhibit a coherent and distinct relationship from a set of attributes terms, and proposed specific methods for debiasing the embeddings. Gonen and Goldberg [15] showed that debiasing methods are insufficient and proposed a way to detect implicit biases by clustering the target terms sets. Nikhil et al. [14] studied gender and ethnic biases in the United States for the past 100 years, obtaining evidence that the word embeddings under consideration captured nuances from the women’s liberation movement.

Applying these methods to Arabic word embeddings exhibits a significant challenge. The Arabic language presents unique difficulties in quantifying these biases, including its morphological complexity, where many words can be derived from the same root [4]. Moreover, Arabic words often show various meanings depending on the context in which they are used. To illustrate, the word "رسم" can carry different meanings. In an artistic context, it refers to “drawing" or “artwork". However, in an economic context, it means “fee" or “charge". Similarly, the word "جدة" has different interpretations based on the context in which it is used. In the context of familial relationships, the word signifies “grandmother", whereas in the context of geographical location, it refers to the city “Jaddah", a city in Saudi Arabia. Consequently, the nature of Arabic words introduces ambiguity and obscurity, complicating the interpretation of word embeddings and the process by which individual words can be used to measure bias in Arabic.

Additionally, and unlike English, Arabic is a grammatically gendered language where words can be classified into masculine or feminine. The Arabic word "ممرض", representing “male nurse", is grammatically masculine, and "ممرضة", denoting “female nurse", is grammatically feminine. Due to grammatical gender, Arabic word professions are naturally associated with female and male components in the embedding space, and this association should not be deemed a stereotype [33]. However, gender bias is still present in Arabic word embeddings where, when projecting the male and female professions into the gender subspace, for example, one observes that the word vector "ممرضة" (female nurse) is inclined more towards the female direction than the word vector "ممرض" (male nurse) is towards the male direction, which ideally should be a symmetrical inclination in order to be free of any gender bias [33]. On a more overarching note, this work highlights the importance of unlocking existing bias detection models to make them useful and relevant for societal issues affecting the Arab world, especially those that remain understudied from a Western lens and are not adequately addressed by local societies.

Identifying gender bias in gendered languages using word embeddings has been previously explored to some extent. McCurdy and Serbetci [23] expanded the WEAT tests and incorporated three additional grammatically gendered languages: Dutch, German, and Spanish. Lauscher and Glava [21] also followed the same approach and developed a cross-lingual and multilingual WEAT framework including German, Spanish, Italian, Russian, Croatian, and Turkish languages. Zhou et al. [33] and Sabaghi and Caliskan [29] delved into bias within grammatically gendered languages, revealing that word embeddings encompass both semantic and grammatical gender components, potentially skewing measurements of social gender bias. Zhou et al. [33] introduced MWEAT, a modified version of the WEAT test specifically considering grammatical gendered words, applied to the French and Spanish languages. This authors’ nuanced exploration addresses an essential gap in the literature, paving the way for a deeper understanding of gender bias in languages with grammatical gender components. Sabaghi and Caliskan [29] further proposed methods to assess, disentangle, and evaluate grammatical gender signals quantitatively. Lauscher et al. [22] examined biases within Arabic word embeddings. Their investigation spans various dimensions, consisting of different factors such as the characteristics of the embedding models and vector sizes, the diverse nature of text sources, the differentiation between dialects, and a temporal analysis involving corpora from distinct historical periods. Studies have further expanded the scope of bias analysis in natural language processing. Wallace and Paul [30] uncovered implicit stereotypes in user-generated content such as online reviews, highlighting the importance of examining diverse data sources. Font and Costa-jussà [13] conducted a comprehensive study on gender bias in English, Spanish, and German word embeddings, providing a valuable framework for cross-linguistic analysis. This work, along with studies by Rudinger et al. [28], demonstrated that biases in word embeddings can significantly impact downstream NLP tasks. The implications of these biases extend beyond language models to information retrieval systems. Kay et al. [19] showed how web search results can be biased, showing the need to investigate gender bias in search systems across languages.

In this manuscript, we propose different methods for evaluating bias in Arabic word embeddings, and gendered languages in general. Our contribution can be summarized as follows:

1. We generate a list of word pairs that can be used in the Direct Bias method to represent the gender direction. Since our focus in the Direct Bias method is on occupations, for each candidate pair representing gender, for example, $\vec{she}$ (هي) and $\vec{he}$ (هو), we state that this pair ($\vec{she}$, $\vec{he}$ ) i.e. (هو هي،) accurately represents the gender direction when the female occupation vector is closer to $\vec{she}$ (هي) than $\vec{he}$ (هو), and simultaneously the male occupation form is closer to $\vec{he}$ (هو) than $\vec{she}$ (هي).
2. We generate a list of word pairs representing the male and female versions of various professions and we extend the Direct Bias method to be able to quantify bias using those word pairs representing professions. Our extension involves the assessment of symmetry between two profession forms, masculine and feminine in relation to the gender direction vector identified in the previous step.
3. We suggest a list of proper Arabic words that can be used for the WEAT Test to detect gender bias. The WEAT test allows the study of association of men with science in parallel with an association of women with arts. Our findings show a significant association between men and science, contrasted against a significant association between women and arts, exposing a bias that emphasizes the prominence of men in fields associated with “career".
  We incorporate all the above to investigate gender bias in Arabic text using word embeddings trained on multiple Lebanese news archives, Wikipedia, and different electronic newspapers. We used the Lebanese news archives, which span several decades (1935-2011), to explore the evolving trends in gender bias within the Lebanese culture. Additionally, we examined Arabic Wikipedia data, a rich and comprehensive source that allows us to investigate the gender bias presented in Arabic text and thus overview the whole Arab Region. We also included various electronic newspapers based in the Arab region, specifically focusing on the UAE, Egypt, and Morocco. Our findings reveal consistent gender biases across professions. Even though all the employed datasets revealed a mutual and static, biased perception toward the females in professions such as nurse, house cleaner, maid, secretary, and dancer, the Moroccan newspaper stands out by showing an additional female engagement in other marked professions like researcher, doctor, and professor. On the other hand, the Lebanese news archives and Wikipedia data consistently show bias towards males in professions such as doctor, lawyer, representative/deputy, and writer, while the Moroccan newspaper exhibits a distinct bias towards males in high-level positions including manager, minister, representative, and ambassador. We also explore the evolution of such bias in time in the Lebanese context whenever applicable. As such, our analysis attains synchronic variation analysis (across corpora), and diachronic variation analysis (across time) [24].

Our study addresses several gaps in the existing literature on bias analysis in Arabic word embeddings. While previous research has extensively explored gender bias in various languages, there is an absence of a comprehensive investigation focusing specifically on Arabic, a grammatically gendered language. We introduce Direct Bias and WEAT tests specifically tailored for Arabic and conduct in-depth analyses that provide valuable insights into gender biases. Furthermore, our work emphasizes the need for linguistic sensitivity. Given the complexities of Arabic, we propose measures to ensure that our test specifications are contextually appropriate. For example, in the direct bias approach, we suggest a methodology to choose the proper female/male word pairs reflecting the gender context and propose an extension of the bias computation to address gendered word forms. Furthermore, we customize the English WEAT tests for application in an Arabic context due to its utility in representing gender bias associations. In tandem with the related work surveyed in the literature above, our research extends the scope of bias analysis by enclosing a more comprehensive and detailed examination of gender biases in Arabic word embeddings through presenting gender bias across various occupations and exploring the association of women with arts and domestic responsibilities in parallel with the association of males in science and career, thereby filling significant gaps in the associated literature.

## Materials and methods

### Methodology overview

**Fig 1:**
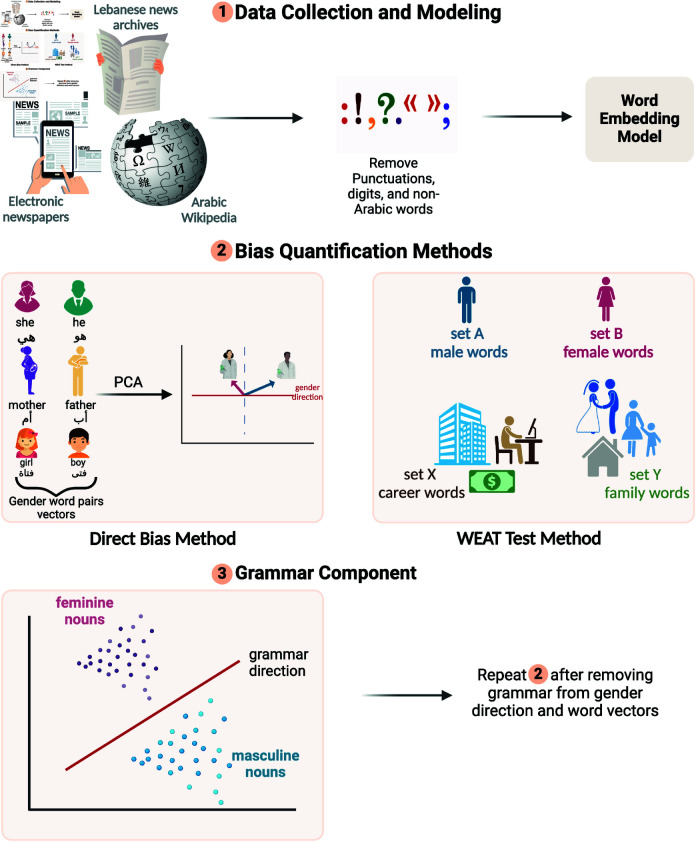
Schematic overview of the data and methodology used for quantifying gender bias.

Fig 1 presents a schematic overview of our research methodology for analyzing gender bias in Arabic text through word embeddings. The code implementing our methodology is publicly available [26] with all the statistics plotted in this manuscript. Our approach consists of three main stages: (1) Data Collection and Modeling, (2) Bias Quantification Methods, and (3) Grammar Component analysis.

In the first stage, we collect data from various Arabic text sources, including news archives and Wikipedia, then preprocess it by removing punctuation and non-Arabic words before training our word embedding model.

The second stage employs two primary methods for quantifying gender bias: the Direct Bias Method and the WEAT Test Method. For the Direct Bias Method, we first carefully selected Arabic word pairs that accurately represent gender concepts, such as هي/هو (she/he) and امرأة/رجل (woman/man). To accurately identify the gender subspace in Arabic word embeddings, we developed a novel classification approach tailored to the complexities of the Arabic language. We began by compiling an extensive list of potential gender word pairs (Table 1) and occupation terms (Table 2) in masculine and feminine forms. For each candidate pair, we calculated the cosine distance between the pair terms and various occupation vectors. A pair was considered suitable for representing the gender direction if the female term was consistently closer to female occupation vectors and the male term to male occupation vectors. We set a threshold of 75% consistency across occupations for a pair to be included in our final set. This method ensured that our selected word pairs robustly captured gender direction. The Direct Bias Method uses Singular Value Decomposition (SVD) on a matrix of differences between male and female word pairs to derive a gender direction vector $\vec{d_{g}}$. We adapted this method for Arabic by extending the bias calculation to account for gendered noun forms (Eq 6), primarily applying it to analyze occupational words and quantify gender bias in professional contexts.

**Table 1:** Female and male word pairs.

Word Pairs
ابنة (Daughter), ابن (Son), أمومة (Motherhood), أبوة (Fatherhood), أخوات (Sisters), أخوة (Brothers), أرملة (Widow), أرمل (Widower), زوجات (Wives), أزواج (Husbands), أمهات (Mothers), آباء (Fathers), أم (Mother), أب (Father), أمي (My mother), أبي (My father), عمات (Aunts), أعمام (Uncles), اناث (Females), ذكور (Males), عمة (Aunt), عم (Uncle), أميرة (Princess), أمير (Prince), حفيدة (Granddaughter), حفيد (Grandson), راهبة (Nun), راهب (Monk), أنثى (Female), ذكر (Male), أخت (Sister), أخ (Brother), سيدات (Ladies), سادة (Gentlemen), نساء (Women), رجال (Men), حفيدات (Granddaughters), أحفاد (Grandsons), سيدة (Lady), سيد (Sir), عروس (Bride), عريس (Groom), عشيقة (Female Lover), عشيق (Male Lover), ملكات (Queens), ملوك (Kings), صديقة (Female Friend), صديق (Male Friend), امرأة (Woman), رجل (Man), لها (Her), له (His), نفسها (Herself), نفسه (Himself), هي (She), هو (He), ملكة (Queen), ملك (King), أميرات (Princesses), أمراء (Princes), فتاة (Girl), فتى (Boy), فتيات (Girls), فتيان (Boys), زوجة (Wife), زوج (Husband), شابة (Young woman), شاب (Young man), ماما (Mom), بابا (Dad), جدات (Grandmothers), أجداد (Grandfathers), بنات (Daughters), أبناء (Sons), جدة (Grandmother), جد (Grandfather)

**Table 2:** Female and male occupations.

Occupation
Engineer: مهندسة مهندس , Doctor: طبيبة, دكتورة طبيب, دكتور , Lawyer: محامية محامي , Police Officer: شرطية شرطي , Nurse: ممرضة ممرض , Seller: بائعة بائع , Pharmacist: صيدلانية صيدلي , Employee: موظفة موظف , Manager: مديرة مدير , Writer: كاتبة, أديبة كاتب, أديب , Researcher: باحثة باحث , Journalist: صحفية صحفي , Ambassador: سفيرة سفير , Minister: وزيرة وزير , Servant: خادمة خادم, Cook: طباخة طباخ , Secretary: سكرتيرة سكرتير , Deputy: نائبة نائب , Professor: بروفيسورة بروفيسور, Teacher: أستاذة أستاذ , Photographer: مصورة مصور, Driver: سائقة سائق, Dancer: راقصة راقص , Singer: مغنية مغني , Actor: ممثلة ممثل , Artist: فنانة فنان , Poet: شاعرة شاعر , Baker: خبازة خباز , Worker: عاملة عامل , Gardener: بستانية بستاني , Janitor: ناطورة ناطور, Inspector: مفتشة مفتش , Musician: عازفة عازف , Soldier: جندية جندي , Guard: حارسة حارس , Painter: رسامة رسام

**Table 3:** WEAT tests stimuli.

	Attribute1	Attribute2	Target1	Target2
**Test1:** flowers-insect	Pleasant سرور (joy), جميل (beautiful), لطيف (kind), هادئ (calm), رائع (wonderful), سلام (peace), سعيد (happy), مريح (comfortable)	UnPleasant مؤلم (painful), قتل (killing), غاضب (angry), إساءة (abuse), حزين (sad), مخيف (scary), مزعج (annoying), موت (death)	Flowers ياسمين (jasmine), زنبق (lily), زهرة (flower), وردة (rose), ليلك (lilac), أقحوان (chrysanthemum), بنفسج (violet)	Insects نملة (ant), ذبابة (fly), حشرة (insect), نحلة (bee), فراشة (butterfly), بعوضة (mosquito), صرصور (cockroach)
**Test2:** music-weapon	Pleasant	UnPleasant	Musical Instruments مزمار (flute), العود (oud), طبل (drum), قيثارة (lyre), ناي (reed flute) الكمان (violin), الساكسفون (saxophone), بيانو (piano), تشيلو (cello) جيتار (guitar)	Weapons خنجر (dagger), سيف (sword), بندقية (rifle), مسدس (pistol), قنبلة (bomb), السكين (knife), المدفع (cannon), صاروخ (missile), فأس (axe)
**Test3:** career-family	Career الوظيفة (job), مهنة (career), الشركة (company), راتب (salary) مكتب (office), المؤسسة (institution), الإدارة (management), عمل (work)	Family عائلة (family), منزل (home), أبوين (parents), أطفال (children), أبناء (sons) زفاف (wedding), أقارب (relatives), بيت (house), زواج (marriage)	Male Names يوسف (Yusuf), محمد (Mohammed), أحمد (Ahmed), علي (Ali) طارق (Tariq), رامي (Rami), مصطفى (Mustafa), أيمن (Ayman)	Female Names ليلى (Leila), فاطمة (Fatima), سلمى (Salma), نور (Noor) هدى (Huda), رنا (Rana), زينب (Zainab), مريم (Maryam)
**Test4:** math-art	Male terms الجد (grandfather), شقيق (brother), له (his), إبن (son), صبي (boy) هو (he), الذكور (males)	Female terms أنثى (female), فتاة (girl), هي (she), إبنة (daughter), شقيقة (sister), نساء (women) لها (hers)	Math الطرح (subtraction), إحداثيات (coordinates), إحصاء (statistics) معادلات (equations), رياضيات (mathematics), مجموع (sum) أعداد (numbers), حساب (calculation), أرقام (digits)	Art رواية (novel), غناء (singing), لوحة (painting), سينما (cinema), مسرح (theater) موسيقى (music), رقص (dance), فنون (arts)
**Test5:** science-art	Male terms	Female Terms	Science رياضيات (mathematics), الطب (medicine), علوم (sciences), فيزياء (physics) الابحاث (research), المختبرات (laboratories), كيمياء (chemistry)	Art
**Test6:** intellects-appearance	Intellects النضوج (maturity), المهارة (skill), الإبداع (creativity), الخبرة (expertise) العبقرية (genius), الفطنة (acuity), الحدس (intuition), الحكمة (wisdom) المنطق (logic), الذكاء (intelligence), التحليل (analysis)	Outer appearance البشاعة (ugliness), البدانة (obesity), النحافة (thinness) الأناقة (elegance), الوسامة (handsomeness), الجاذبية (attractiveness) الضعف (weakness), الجمال (beauty)	Male Names	Female Names

The WEAT Test Method examines more nuanced associations between broader concepts, investigating how gender stereotypes manifest in relation to semantic categories such as career vs. family, science vs. arts, and intellect vs. appearance (Table 3). This method measures the differential association between sets of target words (e.g., career-related, family-related) and attribute words (e.g., male, female). We calculated the test statistic s(X,Y,A,B) as shown in , which quantifies the difference in associations between two sets of target words X and Y with respect to two sets of attribute words A and B. Additionally, we computed Cohen’s effect size d (Eq 10) to quantify the magnitude of bias, providing a standardized measure of the strength of associations. This approach allows us to capture stereotypes beyond just occupations, offering insights into broader gender-based associations in Arabic text. By applying WEAT across various semantic categories, we can uncover subtle biases that might not be apparent in occupation-specific analyses.

In the third stage, we address the challenges posed by Arabic’s grammatical gender system. We train a classifier to identify the grammatical gender signal ${\vec{d}}_{gr}$, which we then remove from both individual word vectors (Eq 11) and the gender direction (Eq 12). This grammatical gender disentanglement process allows us to distinguish semantic gender bias from bias inherent in the language’s structure. We repeat the bias quantification methods after this grammatical component removal to ensure our analysis captures true semantic biases rather than artifacts of Arabic’s grammatical structure.

Our goal from employing the datasets is to study to what extent these textual corpora reflect the already established gender bias in the Arab region, and to compare such bias across corpora, and across time when applicable. We examined occupations that are culturally significant in Arab societies, such as engineer (مهندس-مهندسة), teacher (معلم-معلمة), and nurse (ممرض- ممرضة), as well as modern professions and leadership roles. This selection allows us to investigate how traditional gender roles and evolving societal norms are reflected in language use across different domains and time periods in the Arab world. Combining the Direct Bias analysis of specific occupations with the broader conceptual associations revealed by WEAT, we aimed to provide a comprehensive picture of gender bias as reflected in Arabic text.

### Data collection and modeling

#### Data collection.

**Lebanese news archives:** Our first two datasets were obtained from two prominent Lebanese news archives, namely Annahar and Assafir, which were obtained as OCR’ed text from images corresponding to microfilms from the American University of Beirut Libraries [8]. The collection and use of these datasets complied with the copyright and term of use instructions published here. Specifically, because the AUB is a third-party organization and legally restricts the sharing and copying of the actual data sets onto external public repositories, we have instead provided a minimal dataset consisting of the word embeddings trained on these archives [32]. Assafir, which ceased operations in December 2016, contributed an archive comprising 12,058 issues spanning 1974 to 2011, covering 37 years. Annahar’s news archive spans a more extensive period, from 1933 to 2009, encompassing 23,112 issues with a range of 76 years. These archives span several decades, allowing for diachronic (temporal) analysis of gender bias evolution.

**Wikipedia:** Our third dataset was obtained from the Arabic Wikipedia, a publicly available dataset. Wikipedia contributes to over 45 million categorized articles in 285 languages, including Arabic. Notably, Arabic became the first Semitic language to surpass 100,000 Wikipedia articles. To support diverse applications, Wikipedia offers freely accessible database copies. For our models, we obtained the latest Arabic dump, dated June 1, 2023 [31]. We further segmented 8,111,436 pages into paragraphs, each serving as a document for constructing our model. The use of Wikipedia data complies with the Creative Commons Attribution-ShareAlike 3.0 Unported License (CC BY-SA 3.0) and the GNU Free Documentation License (GFDL).

**Electronic newspapers:** Our final datasets were two publicly available datasets obtained from electronic Arabic newspaper archives:

- The Ultimate Arabic News Dataset [1], a comprehensive collection of modern Arabic texts sourced from reputable news websites such as Al-Arabiya located in the UAE and Al-Youm Al-Sabea (Youm7) located in Egypt. We used the unprocessed “UltimateArabic" file containing over 193,000 original news texts. The use of this dataset complies with the terms specified by the dataset creators for research purposes.
- The Moroccan News Articles Dataset (MNAD) [18], with Version 1 comprising 418,563 categorized Moroccan news articles and Version 2 (MNAD.v2) expanding to 1,069,489 articles by incorporating data from seven additional prominent Moroccan news websites. Our use of the MNAD dataset adheres to the terms set by the dataset creators for academic and non-commercial research activities.

**Data preprocessing:** We refrained from applying normalization to the Arabic word embeddings to preserve the original Arabic text and its meaning, because of the following reasons. Normalization in Arabic, such as unifying similar letters (e.g., replacing Ta Marbouta ("ة") with Ha ("ه"), converting "أ" (Hamza above Alef), "آ" (Hamza with Madda above Alef), and "إ" (Hamza below Alef) to bare Alef ("ا"), or replacing "ى" (Alef Maqsura) with "ي" (Ya)), risks conflating distinct words that are semantically and syntactically different. For example, "طبيبة" (female doctor) could become "طبيبه" (his doctor), causing us to lose the gender-specific distinction for occupation bias, and for the word embedding to represent both words using the same vector. Similarly, the word pair "ابنة" (daughter) could be conflated with "ابنه" (his daughter), making it challenging to find word pairs for the direct bias method and to preserve the contextual meaning. The word "على" (on/upon) could be normalized to "علي" (a common male name, Ali), changing its interpretation entirely and adding challenges to the WEAT test. Most of the Arabic text utilized in our case studies did not include diacritics, and our pre-processing primarily involved removing punctuation marks (e.g., “.", “,", “?", "!"), digits, and non-Arabic words, while avoiding any modifications that might alter the underlying meaning of the text.

#### Word embedding model.

We employed the continuous bag-of-words (CBOW) model [25] as the foundation for our embeddings. The CBOW model is a neural network-based approach that predicts a target word based on its surrounding context, without considering word order. The model aims to create dense vector representations of words by learning from large text corpora. Mathematically, For a sequence of words $w_{1},w_{2},\ldots ,w_{T}$, the objective of CBOW is to maximize the average log probability:

1T∑t=1Tlog ⁡p(wt∣wt−c,… ⁡,wt−1,wt+1,… ⁡,wt+c) (1)

where *c* is the size of the context window. The probability $p(w_{t}|\text{context})$ is computed using the softmax function:

$$\begin{array}{ccc} p(w_{t}|\text{context})=\frac{\text{exp}\left({v_{w_{t}}^{\prime }}^{⊤}{\bar{v}}_{c}\right)}{\underset{w\in W}{\sum }\text{exp}\left({v_{w}^{\prime }}^{⊤}{\bar{v}}_{c}\right)} & & \end{array}$$ (2)

where $v_{w_{t}}^{\prime }$ and $v_{w}$ are the ’output’ and ’input’ vector representations of *w*, *W* is the vocabulary, and ${\bar{v}}_{c}$ is the average vector of the context words. This model architecture efficiently captures semantic similarities by learning to predict words in a given context. For the Lebanese news archives, the word embeddings were trained at a yearly level to study the evolution of gender bias across time. For all the other datasets, the word embeddings were trained on the whole dataset. All word embeddings were trained using Gensim, an NLP toolkit that includes a Word2Vec implementation with a window size of 5, a minimum count threshold of 10, and embedding dimension *d* equal to 300. The training times varied: the Wikipedia and electronic newspapers models took approximately 4 hours each on a Windows 10 PC with an Intel (R) Xeon(R) Gold 5120 CPU @2.20 GHz and 64 GB of RAM, whereas the Lebanese news archive models took around 15-20 minutes per each yearly level to train the respective models.

### Gender bias quantification

Our research focuses on quantifying gender bias in Arabic word embeddings using Direct Bias [3] and WEAT tests [5]. Both methods involve selecting specific words to evaluate bias, with Direct Bias relying on male-female word pairs and the WEAT test utilizing word stimuli for each test. However, our investigations encountered challenges arising from ambiguity in Arabic word embeddings. The absence of diacritics in many Arabic texts and word embeddings trained on them can lead to potential obscurity in word meanings, making it problematic to accurately select appropriate word pairs for bias evaluation. Diacritical marks, in general, are symbols that accompany letters and serve to modify their sounds or differentiate words from homonyms. These marks are common in Semitic languages like Arabic, Hebrew, and Urdu [6]. Their primary objective is to clarify various linguistic and phonetic aspects, such as the morphological structure, grammatical function, and semantic meaning of words. In contrast to Latin languages like French, where vowels are always present in texts, Arabic texts often lack diacritical marks. Boudchiche and Mazraoui [4] indicate that diacritical marks are missing in approximately 98% of Arabic texts, resulting in considerable potential ambiguity in over 77% of cases [4].

Moreover, researchers often remove diacritics for various reasons. One such reason is to reduce dimensionality. For instance, consider a sentence "جميل الكتاب", meaning "The book is beautiful". With diacritics, it is written as "جَمِيلٌ الكِتَابُ". However, for embedding purposes, researchers may opt to remove diacritics and represent it as "جميل الكتاب", simplifying the representation without losing the contextual meaning. Another essential advantage of removing diacritics is the ability to create normalized representations for Arabic words. This normalization allows for better comparison and matching of words in downstream Natural Language Processing (NLP) tasks. For example, the word "شركة" means "company," and with diacritics, it is written as "شِرْكَةٌ". By removing the diacritics, the representation becomes "شركة", making it more adaptable for various NLP applications. However, it is essential to state that diacritic removal comes with challenges. One major challenge is the potential ambiguity that arises without diacritics [4], making distinguishing between words with similar spellings or pronunciations tricky. For example, the word "ذكر" without diacritics can be interpreted as “male," and its diacritic representation is "ذَكَر". However, it could also represent another noun "ذِكْر" meaning quoting or citing, and as a verb word "ذَكَرَ" means "he mentioned". Similarly, the word "رجل" conveys in the gender context a man "رَجُلْ". Yet, it could also mean “leg", and its diacritic representation is "رِجْل".

Given these challenges, we propose a novel method to address the issue of ambiguity and choose suitable word pairs and stimuli for effectively conducting the Direct Bias and WEAT tests. Our approach aims to choose valid words to ensure a reliable evaluation of gender bias in grammatically gendered embeddings, specifically Arabic.

#### Direct bias.

We follow the methodology proposed by Bolukbasi et al. [3]. We begin with a word embedding $W\in {\mathbb{R}}^{d}$ and word-pair sets $P_{1},P_{2},...,P_{n}\subset W$ (e.g., $P_{1}=\{\vec{she},\vec{he}\}$, $P_{2}=\{\vec{her},\vec{him}\}$, $P_{3}=\{\vec{mother},\vec{father}\}$, etc.). Each word pair consists of two words: one represents the female gender, and the other is associated with the male gender, thus symbolizing both genders. This pairing approach helps us derive the gender direction vector ($\vec{d_{g}}$) within the word embedding matrix *W*. For each word-pair set $P_{i}\in P_{1},P_{2},...,P_{n}$, we perform the following steps:

1. Let $u_{i}$ denote the centroid of the word pairs in the set $P_{i}$ (e.g $P_{1}\{\vec{she},\vec{he}\}$) where
  $$\begin{array}{ccc} u_{i}=\frac{\underset{p\in P_{i}}{\sum }\vec{p}}{\left|P_{i}\right|} & & \end{array}$$ (3)
2. We calculate and include in the matrix *C* the covariance value for each word *p* in the set $P_{i}$
  $$\begin{array}{ccc} \frac{\underset{p\in P_{i}}{\sum }{\left(\vec{p}-u_{i}\right)}^{T}(\vec{p}-u_{i})}{\left|P_{i}\right|} & & \end{array}$$ (4)

We then utilize the Singular Value Decomposition (SVD) technique on the covariance matrix *C* resulting from the steps mentioned using all word pairs to identify the bias subspace *B*. SVD decomposes the matrix *C* into three fundamental matrices $U\Sigma V^{T}$, where the eigenvectors are represented by matrix *U*. In this context, the bias subspace *B* corresponds to the first row of the matrix *U*, which captures the principal component with the highest variance, detecting the gender direction ($\vec{d_{g}}$) in the data associated with the word pairs.

**Gender subspace identification:** Arabic is a complex language with rich morphology, where words undergo various transformations based on their roles in sentences. Identifying relevant word pairs is crucial for studying gender biases. Additionally, words’ meanings can differ significantly depending on the context in which they appear, making it essential to capture these contextual nuances when selecting word pairs for analysis. Thus, we propose a classification task for grammatically gendered languages such as Arabic to choose proper word pairs that accurately represent the gender subspace.

Consider the list of word pairs, as shown in Table 1, and the list of female and male occupations, as displayed in Table 2. For each word pair in the list, such as {ابنة (Daughter), ابن (Son)},{أمومة (Motherhood), أبوة (Fatherhood)},{أخوات (Sisters), أخوة (Brothers)}, etc., we calculate the distance between the word pair and each female occupation term, like مهندسة (female engineer), طبيبة (female physician), دكتورة (female doctor), etc. If the female occupation term is closer to the female word pair term than its male counterpart, for instance, if مهندسة (female engineer) is closer to ابنة (daughter) than to ابن (son), we consider that word pairs, e.g. {ابنة (Daughter), ابن (Son)}, as suitable for representing the gender direction for females. We repeat this process for male occupation terms, such as مهندس (male engineer), طبيب (male physician), دكتور (male doctor), etc., and calculate the distances between each male occupation term and both male and female word pair terms. If the male occupation term is closer to the male word pair term than the female one, for instance, if مهندس (male engineer) is closer to ابن (son) than to ابنة (daughter), we consider that candidate pairs, e.g. {ابنة (Daughter), ابن (Son)} suitable for representing the gender direction for males.

Applying this approach to all the word pairs, we set a threshold of 75% for which a word pair is considered suitable for representing each gender direction. This means that a word pair needs to satisfy our criteria in at least 75% of the cases; for example, male pairs should be closer to at least 75% of the male occupation vectors than the female pairs, and conversely, female pairs should be closer to at least 75% of the female occupation vectors than the male pairs.

After conducting these analyses, we obtained two distinct lists: one containing suitable candidate pairs representing the male gender direction and another comprising appropriate candidate pairs representing the female gender direction. We take the intersection of the two lists to ensure that our word pairs robustly capture both male and female directions. The resulting list encloses word pairs that demonstrate the gender subspace. In Table 4, we provide an example of final candidate word pairs using one of the embedding models used in our case studies. For instance, considering the word pair containing the male term صديق (male friend) and the female term صديقة (female friend), we observe that the male pair is closer than the female pair to 93% of the male occupations.

**Table 4:** Word pairs used to define gender direction.

Female Pair	Male Pair	% of Male occupations	% of Female Occupations
صديقة (female friend)	صديق (male friend)	93%	89%
امرأة (woman)	رجل (man)	85%	78%
لها (her)	له (his)	81%	96%
نفسها (herself)	نفسه (himself)	96%	96%
هي (she)	هو (he)	93%	85%
فتاة (girl)	فتى (boy)	85%	100%
شابة (young woman)	شاب (young man)	96%	85%

**Direct bias quantification:** To quantify gender bias in English embeddings, Bolukbasi et al. [3] propose the following methodology: given a word $\vec{w}\in W$ and the gender direction learned as mentioned earlier ($\vec{d_{g}}$), we define the direct gender bias as follows:

$$\begin{array}{ccc} DirectBias=cos(\vec{w},\vec{d_{g}}) & & \end{array}$$ (5)

We extend the definition to quantify gendered word forms in gendered languages. For nouns with masculine ($\vec{w_{m}}$) and feminine ($\vec{w_{f}}$) forms, we assess whether these forms exhibit symmetry with respect to the gender direction:

$$\begin{array}{ccc} DirectBias=|cos(\vec{w_{m}},\vec{d_{g}})|-|cos(\vec{w_{f}},\vec{d_{g}})| & & \end{array}$$ (6)

The direct bias value directly corresponds to the level of gender bias observed. A positive value indicates a bias toward males, a negative value indicates a bias toward females, and a zero value suggests neutrality. For example, suppose the term "دكتور" ($\vec{w_{m}}$) (male doctor) is inclined more towards the male direction compared to the leaning of "دكتورة"($\vec{w_{f}}$) (female doctor) towards the female side. In that case, the direct bias of "دكتور" (male doctor) and "دكتورة" (female doctor) exhibits a positive value, i.e., doctor profession is biased towards males.

#### WEAT test.

Another common approach to quantify bias in word embeddings is the Word Embedding Association Test (WEAT). This method evaluates social stereotype associations present within word embeddings. Drawing inspiration from the Implicit Association Test (IAT) [16] used in social psychology to assess implicit biases in human cognition, WEAT provides a valuable tool for analyzing biases within linguistic data.

During the IAT, participants are presented with two sets of target concepts, such as “science" and “arts", and two sets of attributes, such as “male" and “female". The concepts are further illustrated through stimuli like “physics" and “math" for science-related concepts and “mother" and “girl" for female-related attributes. Participants are then asked to efficiently pair the concepts with the attributes, reflecting their implicit associations. The reaction time in this pairing task quantifies the implicit biases exhibited by the participants. Typically, individuals pair words they find similar more quickly, such as “physics: he" and “music: she". Consequently, the difference in response times between stereotype-congruent (e.g., “science: male" and “arts: female") and stereotype-incongruent (e.g., “science: female" and “arts: male") allows for the computation of an effect size measurement (Cohen’s effect size *d*) for implicit biases. Likewise, the WEAT approach enables the measurement of the differential association between two sets of target concepts and two sets of attributes within word embeddings.

Let *X* and *Y* be two sets of target words, and *A* and *B* be two sets of attribute words. We define the test statistic to quantify the differential association between the target sets and the attribute sets as follows:

$$\begin{array}{ccc} s(X,Y,A,B)=\underset{\vec{x}\in X}{\sum }s(\vec{x},A,B)-\underset{\vec{y}\in Y}{\sum }s(\vec{y},A,B) & & \end{array}$$ (7)

where the association difference of word *w* with attribute sets *A* and *B* and is defined as follows:

$$\begin{array}{ccc} s(w,A,B)=mean_{\vec{a}\in A}cos(\vec{w},\vec{a})-mean_{\vec{b}\in B}cos(\vec{w},\vec{b}) & & \end{array}$$ (8)

The statistical significance of associations in WEAT is calculated through a permutation test, which measures the likelihood of the assumption that an association exists between the targets and the attributes. Utilizing all partitions $\left(X_{i},Y_{i}\right)$ of the union *X*  ∪  *Y* into two sets of equal size, the one-sided *p*-value of the permutation for WEAT can be computed as follows:

$$\begin{array}{ccc} Pr_{i}[s(X_{i},Y_{i},A,B)>s(X,Y,A,B)] & & \end{array}$$ (9)

Cohen’s effect size *d* quantifies the degree to which the association between the targets and attributes is distinct from one another. Thus, a more significant effect size indicates a more substantial bias. Values for *d* exceeding 0.2, 0.5, or 0.8 represent small, medium, and large effect sizes, respectively, and it is computed as follows:

$$\begin{array}{ccc} d=\frac{mean_{\vec{x}\in X}s(\vec{x},A,B)-mean_{\vec{y}\in Y}s(\vec{y},A,B)}{std-dev_{w\in X\cup Y}s(w,A,B)} & & \end{array}$$ (10)

**Tests and lists of stimuli:** To adapt the WEAT tests to the Arabic language, we initially utilized machine translation to automate the translation of the word sets. These word sets were drawn from the target and attribute lists found in the English WEAT tests [5]. After the initial translation, we thoroughly examined the translated words to assess their potential for ambiguity in Arabic. Whenever a translated word could have been associated with multiple meanings or interpretations, we replaced the word with a synonym reflecting the same concept. For instance, suppose the English WEAT test includes the word “poem", which can be translated into Arabic as "شعر"; however, this Arabic word can also mean “hair" and as a verb it means “he felt". Given this ambiguity, we carefully consider the context and replace such ambiguous terms with words that have precise, unambiguous meanings or can be more precisely represented in Arabic. In this context, the word "شعر" was replaced by "فنون". Also, we replaced the male and female name sets with the most prominent Arabic names.

Moreover, given the grammatical gender structure of the Arabic language, we ensured that both genders were considered throughout the translation process by using grammatically gender-neutral words to present each concept, which helps prevent any unexpected bias in our test definitions. To illustrate, the career word sets can be translated into feminine and masculine forms. Thus, we replace them with nouns related to the concept of career, such as “job", “company", “salary", “office", “organization", and “work".

Additionally, we introduce a new test, and we call it *intellects-appearance* test. Under the intellect attributes falls a collection of nouns such as: maturity, skills, creativity, experience, genius, intuition, wisdom, logic, and intelligence. The outer appearance attributes consist of nouns such as ugliness, obesity, thinness, elegance, handsomeness, and attractiveness. We use the *intellect-appearance* test to investigate the association between intellectual attributes with males and physical appearance with females. Table 3 lists the word stimuli used in our WEAT tests.

#### Grammatical gender identification.

Our hypothesis suggests that word embeddings not only capture semantic gender information but also learn a grammatical gender component, as proposed in [29,33]. As a result, we focus on identifying any potential signs of grammatical gender within the embedding space. To identify the grammatical gender direction, we translate a set of approximately 6,000 common French nouns used in [33] into Arabic. This set comprises 3,000 grammatically masculine nouns and an equivalent set of nouns classified as feminine. However, it is important to note that grammatical gender is not inherently preserved during the translation process. To categorize the resulting translated words into Arabic feminine/masculine nouns, we utilize the CAMeL tool [27], an open-source tool for Arabic NLP in Python, to validate whether the translated nouns exhibit the noun’s part-of-speech (POS) characteristics and classify the noun into its corresponding grammatical gender. We then apply a binary classification task categorizing nouns as grammatically feminine or masculine. The goal is to extract a decision hyperplane directly corresponding to grammatical gender ($\vec{d_{gr}}$). We employed Support Vector Classifier (SVC), a linear classifier, to identify this grammatical gender signal. The model is trained using 6,000 translated nouns comprising grammatically feminine and masculine attributes. The resultant decision hyperplane ($\vec{d_{gr}}$), derived from the classification of these nouns, represents grammatical gender direction.

Our approach for disentangling grammatical gender from embeddings draws inspiration from the work of Zhou et al. [33] and Sabbaghi and Caliskan [29]. We assume that these grammatical gender signals might interfere with the measurements of semantic gender bias. Hence, we disentangle these grammatical gender signals from the embeddings. To assess the impact of the disentanglement process on bias measurements, we employ both the Word Embedding Association Test (WEAT) and the Direct Bias method. This allows us to measure the bias present before and after the disentanglement process and observe how these measurements are affected by removing grammatical gender signals. In the WEAT test, the grammatical gender signal is removed from the embedding

$$\begin{array}{ccc} {\vec{w}}^{\prime }=\vec{w}-<\vec{w},\vec{d_{gr}}>\vec{d_{gr}} & & \end{array}$$ (11)

where $<\vec{w},\vec{d_{gr}}>$ is the inner product of the word vector $\vec{w}$ and the grammatical direction $\vec{d_{gr}}$. while in the Direct Bias test, the signal is projected out from the gender direction $\vec{d_{g}}$, resulting in a semantic direction denoted by $\vec{d_{s}}$

$$\begin{array}{ccc} \vec{d_{s}}=\vec{d_{g}}-<\vec{d_{g}},\vec{d_{gr}}>\vec{d_{gr}} & & \end{array}$$ (12)

where $<\vec{d_{g}},\vec{d_{gr}}>$ is the inner product of the gender direction $\vec{d_{g}}$ and the grammatical direction $\vec{d_{gr}}$.

To summarize, our research aims to quantify and analyze gender bias in Arabic text using word embeddings. We employed two main approaches: Direct Bias and Word Embedding Association Test (WEAT), adapted for the Arabic language. Our methodology consists of data collection, preprocessing, word embedding training, bias quantification, and grammatical gender disentanglement. This comprehensive approach allows us to examine gender bias across various Arabic corpora and time periods.

## Results

We present the results of our gender bias quantification for each of the datasets we collected. The code necessary to replicate our results, along with all the data it was based on are publicly available [26].

### Lebanese news archives analysis

Our first case study focuses on the Lebanese news archives. We study gender bias for different occupations and how it changes over time in the local Lebanese news, providing a historical perspective on bias trends over several decades. To quantify and analyze gender bias, we conducted the adapted Direct Bias and WEAT tests that we described in the previous section.

**Fig 2:**
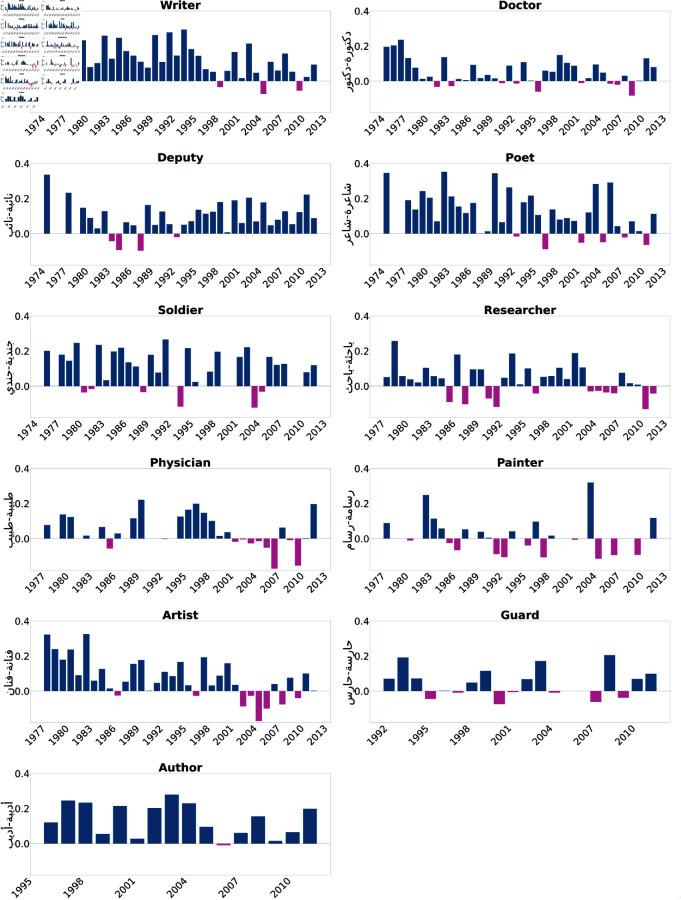
Assafir occupations biased toward males in most of the studied years. Blue bars indicate a positive bias, signifying a preference towards males, while pink bars represent negative values, indicating a bias towards females.

**Fig 3:**
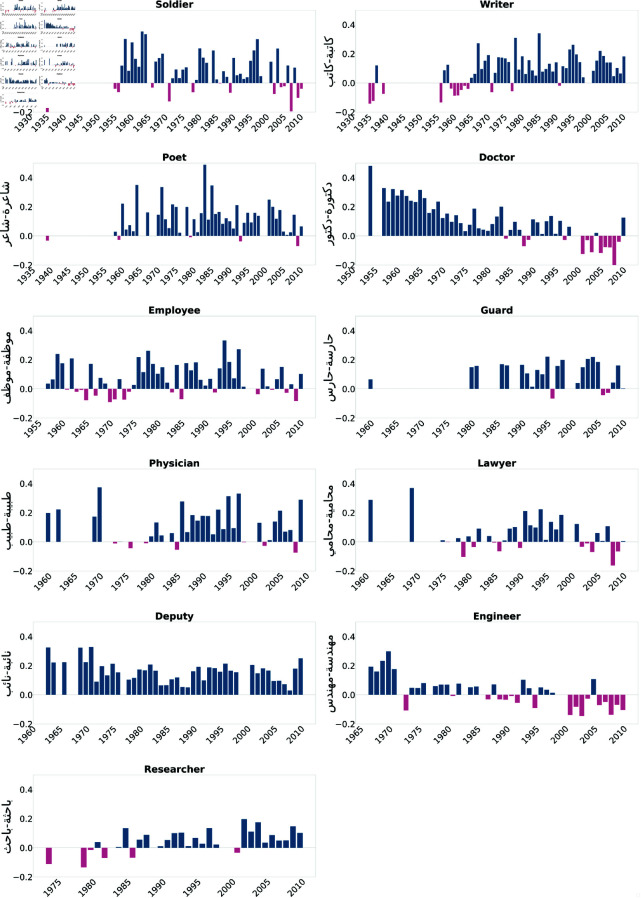
Annahar occupations biased toward males in most of the studied years. Blue bars indicate a positive bias, signifying a preference towards males, while pink bars represent negative values, indicating a bias towards females.

**Fig 4:**
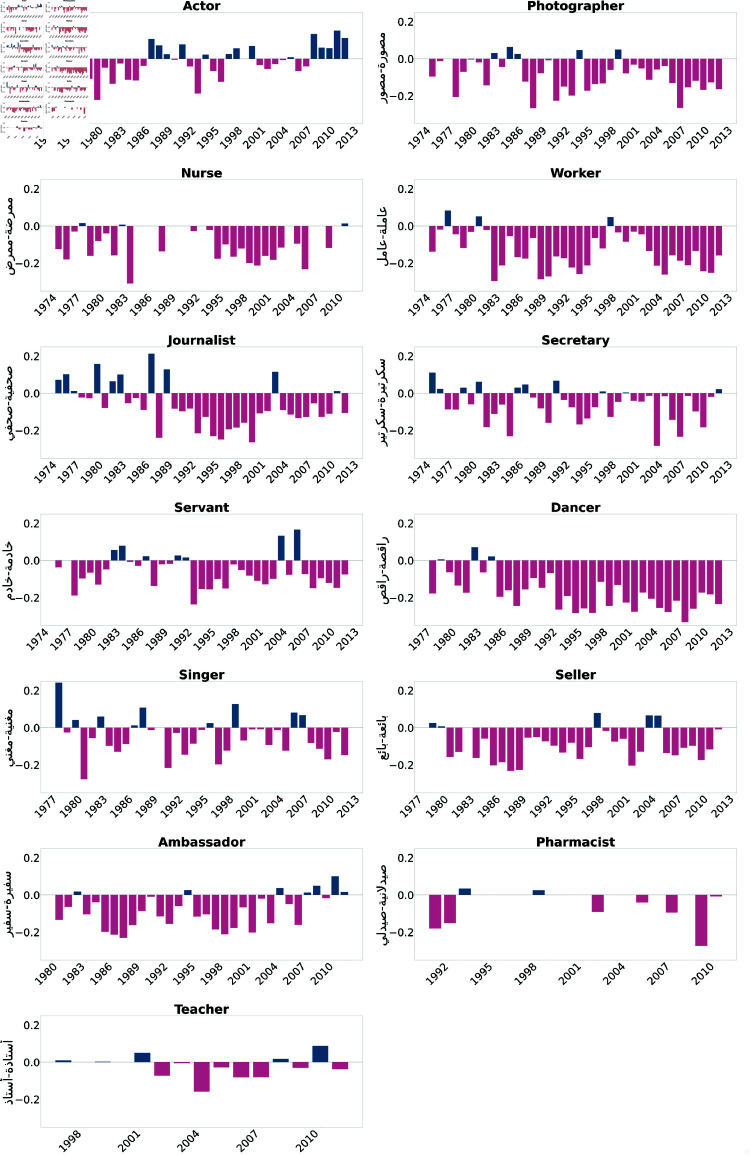
Assafir occupations biased toward females in most of the studied years. Blue bars indicate a positive bias, signifying a preference towards males, while pink bars represent negative values, indicating a bias towards females.

**Fig 5:**
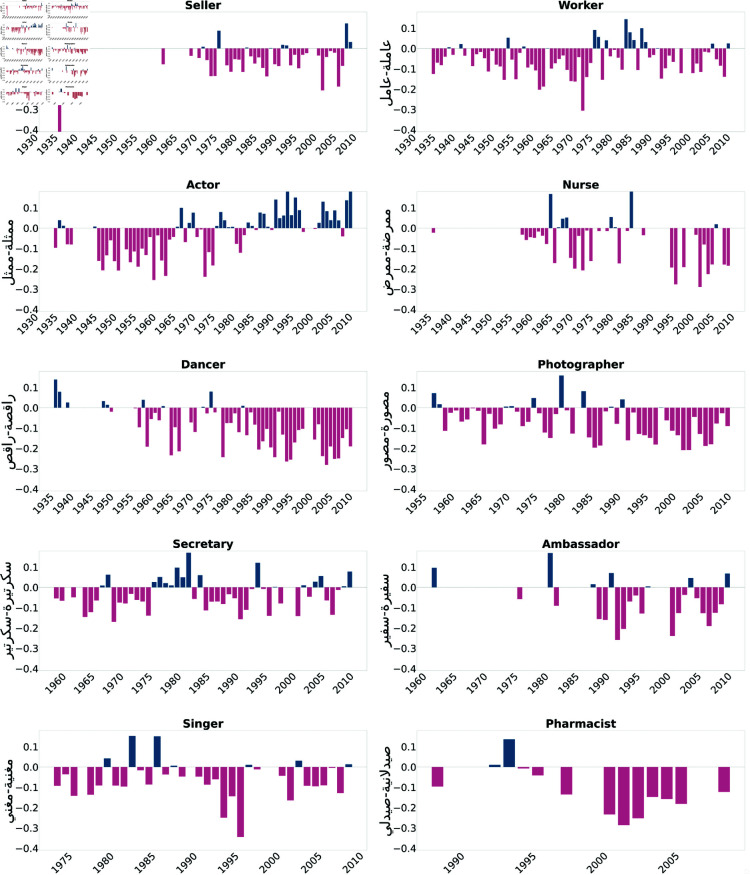
Annahar occupations biased toward females in most of the studied years. Blue bars indicate a positive bias, signifying a preference towards males, while pink bars represent negative values, indicating a bias towards females.

We first present the results of the Direct Bias tests. As illustrated in Figs 2 and 3, professions such as doctor, engineer, lawyer, representative, writer, researcher, artist, novelist, and poet were consistently biased towards males across most of the examined years. Moreover, Figs 4 and 5 show biases towards female-dominated professions, where a persistent female preference was shown in different jobs across the years, including teacher, nurse, house cleaner, maid, secretary, and dancer.

We studied gender bias within the field of medicine, particularly the profession of doctors referred to as "دكتورة-دكتور" (Doctor). The bias within the profession has experienced fluctuations and shifts over the years. Starting from the early 1950s, the bias values in the Annahar newspaper show a consistent positive bias towards males ranging between 0.5 and 0.2, which continued into the mid-1960s. This positive bias trend suggests a historical preference for males within the medical field during this time. However, the values from both newspapers demonstrate that the bias became more subtle in the subsequent years. This bias decreased from the late 1960s to the early 1970s, fluctuating between 0.1 and 0.02, indicating a less consistent gender preference. From the mid-1970s to the early 1990s, however, both newspapers exhibited biased values that mostly favored male doctors. From the mid-1990s to the early 2000s, the bias values show mixed trends, with some years favoring males and others approaching neutrality. In the later years, around the 2000s to the early 2010s, both newspapers showed a bias towards females; however, both sources tended to become positive again, showing the male bias was maintained in 2010 and 2011 with an average score equal to 0.1. We also studied the trends in gender bias in the practitioner/physician profession "طبيبة-طبيب" (Physician), which can also mean doctor in Arabic. This revealed similar patterns to the doctor profession "دكتورة-دكتور" (Doctor). In contrast, the analysis of gender bias trends in the nursing profession "ممرضة-ممرض" (Nurse) for both newspapers demonstrates the bias of this profession towards females. Early through the 1960s, a prevalent negative bias towards female nurses was evident. The bias values onward predominantly favored female nurses, indicating an ongoing preference. Even in recent years, a persisting negative bias has dominated, reflecting constant stereotypes and gender-related expectations within the nursing profession. Similarly, for the pharmacist profession denoted by "صيدلانية-صيدلي" (Pharmacist), a female preference was shown throughout 1995-2000s.

We also examined professions from the literature and news media domain. As an illustration, consider the profession of writers in literature, denoted as "أديبة-أديب" (Author). We observed a consistent tendency toward male writers in the Assafir dataset. Likewise, a similar analysis involves the profession of poets, symbolized as "شاعرة-شاعر" (Poet). In the Annahar dataset, a cosistent series of positive bias values across the years was prominent, reflecting a prevailing inclination towards male poets. This tendency is also echoed in the Assafir dataset, where a consistent positive bias towards males can be noticed over multiple years.

Likewise, the domain of authors, represented by "كاتبة-كاتب" (Writer), demonstrates a matching pattern. A constant bias favoring males emerges when examining both datasets over the years. Whereas the news reporter profession denoted by "صحفية-صحفي" (Journalist) in Assafir data and researcher "باحثة-باحث" (Researcher), reflect a shifting perspective regarding these professions. During the 1970s and 1980s, the bias values revealed a dynamic pattern with a mix of positive and negative trends, indicating a lack of consistent gender bias in favor of either male or female reporters and researchers. However, the 1990s exhibited a distinct negative bias, pointing to an overall preference for female news reporters and a positive bias revealing a preference for male researchers. As we move into the 2000s, a persistent negative bias in the reporting profession endures, varying in intensity from year to year. In contrast, a fluctuating score between positive and neutral was observed in the "researcher" occupation, which suggests an ongoing preference for female news reporters and male researchers, although less pronounced than in previous periods.

Moreover, we examined bias within professions that traditionally emphasize masculinity, focusing specifically on military jobs such as soldiers "جندية-جندي" (Soldier) and security guards “حارسة-حارس" (Guard). Our investigation reveals a prominent gender bias favoring males for both occupations. In the context of security guards, the bias projection shows a progression of values, starting at 0.201 in 1975 and gradually decreasing to 0.08 in 2010, as reported by Assafir. In contrast, a moderate bias persists across Annahar years with an average of 0.2 score. Conversely, in the case of army occupations, the bias projection begins at 0.15 in 1955, peaks at 0.35 in 1962, and goes down to 0.2 in 2008.

The analysis of bias projection within the house cleaner occupation "خادمة-خادم" (Servant) indicates a consistently negative bias over time. A strong perception of female house cleaners was noticed in the 1970s, reflected in values ranging between -0.2 and -0.25. The bias remains predominantly negative across subsequent decades, including notable drops in the late 1980s and early 1990s. Brief positive biases were observed in 2004 and 2006, suggesting potential shifts in perception during those years. However, the bias values overall emphasize enduring societal gender role stereotypes within the occupation. The same pattern was detected in the worker occupation "عاملة-عامل" (Maid), where the plots favor female workers in the field with high peaks of bias reaching -0.35 in 1976 (Annahar) and -0.3 in 1984 (Assafir).

In the domain of art, a significant inclination toward females was observed for the profession of dancing "راقصة-راقص" (Dancer) during the period spanning 1935 to 2011. Similar patterns emerged for the profession of singing "مغنية-مغني" (Singer), with an apparent negative bias evident in the 1980s, where the average bias score stood at -0.3. The bias reached its peak in 1996 with a value of -0.32. In contrast, the occupation of a painter "رسامة-رسام" (Artist) demonstrated an overall bias towards the male gender, peaking at 0.35 in 2004.

When examining the bias scores associated with the occupation of engineers "مهندسة-مهندس" (Engineer) and lawyers "محامية-محامي" (Lawyer) over various years, distinct patterns occur. For engineering, the bias scores indicate a progression from an evident bias towards male engineers in the mid-1960s and 1970s, gradually shifting to a more balanced or even a female-biased representation in the late 20th and early 21st centuries. This suggests a transformation in societal perceptions of gender roles in engineering. On the other hand, the lawyer’s profession shows more varied dynamics. The bias oscillates between a predominantly male preference in certain decades, such as the 1960s, 1980s, and 1990s, and exhibiting a more balanced view in others. We also noticed occasional instances of negative bias, implying a preference for female lawyers, particularly in the mid-2000s.

**Fig 6:**
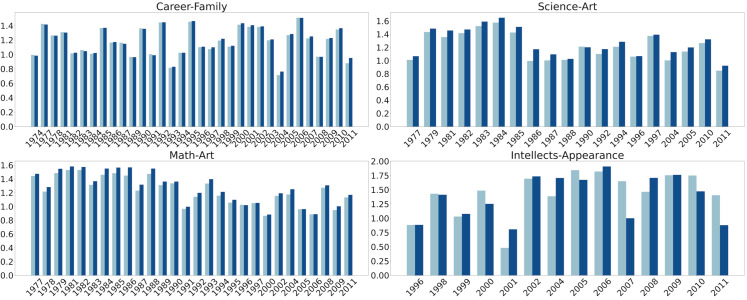
Assafir WEAT effect size comparison before (light blue) and after (dark blue) grammatical disentanglement.

**Fig 7:**
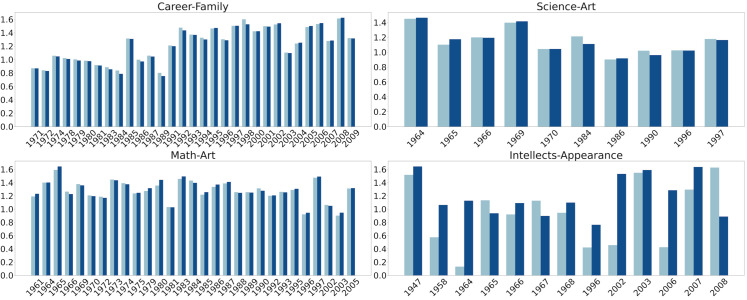
Annahar WEAT effect size comparison before (light blue) and after (dark blue) grammatical disentanglement.

To add, we studied the occupation "نائبة-نائب" (Deputy), meaning "house representative" across different years. The trend reveals a consistently strong positive bias representing the occupation of males, which continued in the early 2000s.

In addition to Direct Bias, we leveraged different WEAT tests across analogous experiments. We present the statistically significant results where the *p*-value is less than 0.05 in Figs 6 and 7. We used the career-family test to examine any affiliation between men and careers, contrasted by a parallel association linking women to familial roles. Our examination extended beyond professional domains to include the associations between mathematical and scientific fields and masculinity, contrasted against the affiliation of artistic disciplines with femininity. We also explored associations that connect intellectual attributes with men and outer appearance with women. The career-family WEAT test applied on both Assafir and Annahar datasets reveals an association between men and careers, and a parallel association correlating women to household responsibilities. The test results in Assafir from 1974 and 2011, and Annahar covering the years from 1971 to 2009, consistently demonstrate biases throughout this period with an effect size greater than 0.8, where career-associated words are strongly associated with men, while family-associated terms are linked to women. Additionally, we employed another WEAT test, named math-art, that focuses on the relationship between math-related words and men, and art-related words and women, covering the period from 1977 to 2011 in Assafir and 1961 to 2005 in Annahar. The *d* values reveal a persistent bias where math-related words are closely connected to men, while art-related terms are correlated with women. Furthermore, we studied the relationship between science-related and art-related words, considering gender bias in these associations (science-art test). A prominent association of science with men and art with women was shown from 1977 to 2011 in Assafir, and a few years in the 1960s, 1980s, and 1990s in Annahar. Throughout this time frame, the *d* values consistently indicate a notable bias, with science-related words more aligned with men and art-related words with women. Moreover, we delved into the relationship between appearance attributes and women, and intellectual attributes and men (intellect-appearance test). In the 1960s, 1990s, and 2000s, a subtle yet statistically significant association emerged between “outer appearance" and women, with an association strength (*d*) value greater than 0.8 for most of the studied years.

### Wikipedia analysis

**Fig 8:**
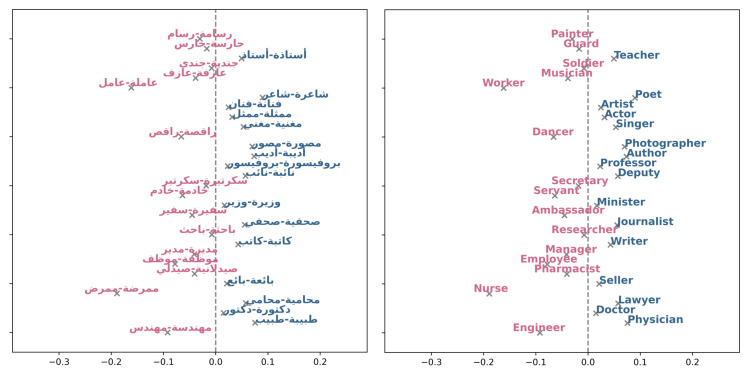
Wikipedia occupations. Blue text indicates a positive bias, signifying a preference towards males, while pink text represents negative values, indicating a bias towards females. The image to the right represents the translation of the words into English, and to the left are the Arabic occupation words.

In addition to traditional news archives, we also set out to study gender bias in Web text by utilizing the Arabic Wikipedia. To this end, we measured the Direct Bias associated with various occupations (see Fig 8). The bias in the figure is represented by a fraction, where a negative ratio means a leaning towards females, while a positive value indicates an inclination towards males. Throughout our analysis, certain occupations that reveal a distinct bias in favor of females emerge, including "ممرضة-ممرض" (nurse), "عاملة-عامل" (worker), "مهندسة-مهندس" (engineer), and "راقصة-راقص" (dancer), all characterized by negative values. This signifies an evident leaning towards females within these roles. In contrast, occupations like "طبيبة-طبيب" (physician), "شاعرة-شاعر"(poet), and "محامية-محامي" (lawyer) yield positive values, indicating a bias towards males in these professional fields. Furthermore, we applied the WEAT tests to the Wikipedia dataset. The results in Table 5 reveal associations between word categories and gender. A significant association between terms like “flower-insect" and “music-weapon" with pleasant and unpleasant categories is noticed as indicated by the effect size *d* (see Table 5) with values greater than 0.8 i.e., 1.543 for “flower-insect" test and 1.05 for the latter. The “career-family" association also displays significant association, as do “math-arts" and “science-arts" (*d* values 1.382, 1.127, and 1.185, respectively), reflecting stereotypes associating women with family and art and men with science, math, and career. Moreover, the “intellects-appearance" category reflects a strong association between females’ outer appearance attributes and males’ intellectualism (*d* 1.48).

**Table 5:** Wikipedia and electronic newspapers WEAT Results: *s* (association strength), *d* (effect size), *p* (p-value), $s_{after}$ (association strength after disentanglement), $d_{after}$ (effect size after disentanglement), $p_{after}$ (p-value after disentanglement), and Δ (change in effect size after disentanglement).

WEAT Test	Dataset	*s*	*d*	*p*	$s_{after}$	$d_{after}$	$p_{after}$	Δ
**flower-insect**	Wiki	0.790	1.543	0.002	0.768	1.525	0.002	–0.018
	UAN	0.281	1.045	0.112	0.280	1.034	0.112	–0.010
	MNAD	0.375	1.318	0.050	0.377	1.316	0.050	–0.002
**music-weapon**	Wiki	0.575	1.050	0.013	0.563	1.049	0.013	–0.001
	UAN	0.484	1.021	0.048	0.480	1.010	0.049	–0.011
	MNAD	0.933	1.416	0.002	0.936	1.416	0.002	0.000
**career-family**	Wiki	0.969	1.382	0.001	1.026	1.411	0.001	0.029
	UAN	1.644	1.623	0.001	1.544	1.600	0.001	–0.024
	MNAD	1.090	1.607	0.001	1.024	1.575	0.001	–0.033
**math-arts**	Wiki	0.525	1.127	0.013	0.575	1.095	0.018	–0.032
	UAN	0.607	1.246	0.008	0.543	1.266	0.006	0.020
	MNAD	0.440	1.115	0.014	0.359	1.114	0.017	–0.001
**science-arts**	Wiki	0.271	1.185	0.012	0.295	1.259	0.004	0.074
	UAN	0.405	1.212	0.012	0.365	1.212	0.011	0.000
	MNAD	0.324	1.019	0.033	0.318	1.055	0.024	0.036
**intellects-appearance**	Wiki	0.476	1.480	0.001	0.439	1.420	0.001	–0.060
	UAN	0.54	1.3122	0.001	0.55	1.3127	0.001	0.0005
	MNAD	0.722	1.562	0.001	0.694	1.570	0.001	0.012

### Electronic newspapers analysis

**Fig 9:**
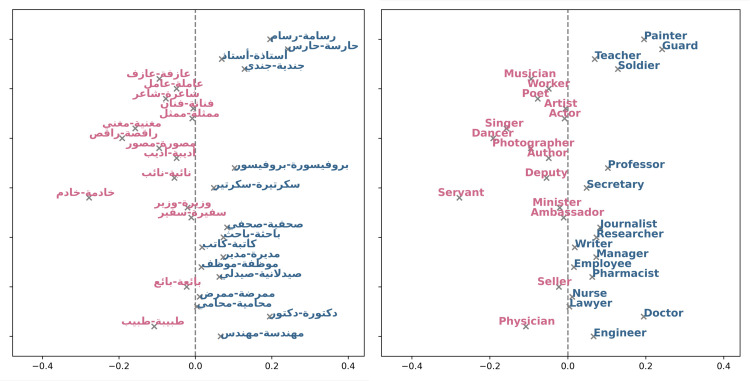
UAN occupations. Blue text indicates a positive bias, signifying a preference towards males, while pink text represents negative values, indicating a bias towards females.

**Fig 10:**
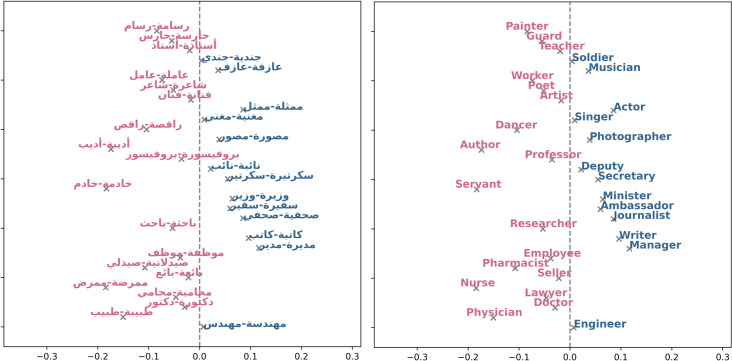
MNAD occupations. Blue text indicates a positive bias, signifying a preference towards males, while pink text represents negative values, indicating a bias towards females.

The Ultimate Arabic News Dataset highlights distinct biases in various occupations with respect to gender (Fig 9). Notably biased towards females are roles such as "خادمة-خادم" (maid/servant), "راقصة-راقص" (dancer), "مغنية-مغني" (singer), and "طبيبة-طبيب" (physician), all of which exhibit significant negative biases. On the other hand, professions like "جندية-جندي" (soldier), "دكتورة-دكتور" (doctor), "رسامة-رسام" (painter), and "حارسة-حارس" (guard) demonstrate substantial positive biases towards males.Upon analysis of the MNAD professions (Fig 10), certain professions exhibit biases. For instance, "ممرضة-ممرض" (nurse), "خادمة-خادم" (maid/servant), "-طبيب طبيبة " (doctor, and "باحثة -باحث" (researcher) show negative values, indicating a bias towards females in these roles. On the contrary, "مديرة-مدير" (manager), "وزيرة-وزير" (minister), "نائبة-نائب" (representative), and "سفيرة-سفير" (ambassador) display positive values, suggesting a bias towards males in high-level positions. We conducted bias analysis shown in Table 5 using the WEAT test on the electronic newspapers datasets. The results reveal consistent gender biases in the word associations being examined, favoring men in the science and career domains and women in the arts and family responsibilities.

### Grammatical gender disentanglement

Finally, we explored the impact of the grammatical gender component on bias measurement. To achieve this, we conducted an analysis using both Direct Bias and the WEAT tests before and after removing the grammatical component from the gender direction and word embeddings, respectively. The occupation results presented previously represent the bias projections achieved after removing grammatical components. We compare the bias before and after eliminating the grammar from the direct bias direction in Table 6. The bias underwent a slight change across the occupations where Δ representing the change of the direct bias across occupations before and after grammatical disentanglement ranged between 0 . 03 and  − 0 . 001. Also, the WEAT test biases are slightly changed after removing grammatical gender associations in all datasets used (see Figs 6, 7, and Table 5). On the other hand, we attempted to explore the impact of grammatical removal on semantics using the music-weapon and flower-insects tests. The Lebanese newspapers archives word embeddings did not match most of the words required for these tests, so we are unable to present any results in this regard. As indicated in Table 5, both tests demonstrated minimal changes, suggesting that grammatical removal did not affect the semantics of Wikipedia and electronic newspapers’ word embeddings.

**Table 6:** Direct bias average values before and after grammatical disentanglement.

	$DirectBias_{before}$	$DirectBias_{after}$	Δ
**Assafir**	–0.0077	–0.0087	–0.001
**Annahar**	0.015	0.014	–0.001
**Wikipedia**	–0.03	–0.004	0.026
**UAN**	–0.01	0.005	0.015
**MNAD**	–0.03	–0.025	0.005

## Discussion

Recent research has been showing a striking gap between women’s improved education and their limited participation in economic activities in several Arab countries spreading from North Africa and to the Middle East [20]. Unemployment rates in the MENA region among young women are nearly 50% higher than among young men. Women in managerial positions are under-represented in the region, with only 11% of women holding managerial positions as compared to the world average of 27.1%. Several factors exist behind the gender disparities that continue to exist in the Arab region today, including within the workforce. As these disparities are attributed to societal gender normal, and whilst we do not attempt to investigate the reasons behind this, our work explores whether such biases are also reflected in several media and online corpora. It takes inspiration from Fairclough and Wodak’s linguistic approach that considers speech - through text – to constitute society and culture, and discourse, to be one of the principles upon which ideology and biases are constructed [11]. To draw the link with elements from critical discourse analysis, our work demonstrated a scalable, automated approach for studying gender-based disparities for occupations by leveraging word embeddings that generalize lexical collocations into the digital sphere by measuring biases using the WEAT and Direct Bias tests on word embeddings. Lexical collocations (or collocational patterns) are based on the intuition that certain words have a tendency to occur near each other in natural language, thereby revealing certain ideological traces and their consequent biases [12]. This is the same intuition by which word embeddings are constructed [3,5,29,33], and bias quantification tests such as the WEAT and Direct Bias tests are designed. Beginning with carefully chosen words representing various occupations, our analysis of prevailing gender stereotypes and prejudices in various professions reflected in word embeddings investigates synchronic variations across the examined corpora for this study, as well as diachronic variations across time in Annahar and Assafir.

At the synchronic level, our comprehensive analysis of gender bias across Lebanese news archives, Wikipedia, and electronic newspapers reveals persistent gender stereotypes in various professions throughout the Arab world. These biases remain evident even after disentangling the grammatical gender component from the word embeddings, suggesting they reflect deeply ingrained societal attitudes rather than artifacts of Arabic’s grammatical structure. Consistently across datasets, females are associated with nurturing and service-oriented roles like nursing and secretarial work, while males are linked to prestigious professions such as medicine and law. The Moroccan newspaper (MNAD), however, showed more female representation in research and academia than in other corpora, while exhibiting male bias in high-ranking positions including management, ministerial roles, parliamentary representation, and ambassadorial appointments. WEAT test results further corroborate these findings, demonstrating strong associations between males and concepts related to science, math, and careers, while females are consistently linked with family, arts, and appearance across all corpora. These associations persisted with only minor changes after removing the grammatical gender component, indicating that the observed biases are not simply a product of Arabic’s gendered language system. In general, and despite marginal differences across the examined corpora, the synchronic trends can be corroborated against existing research indicating that vulnerable or informal, low paying, low-quality employment is particularly high among women in the Arab world, and that women face significant hurdles when entering the most economically secure positions [2,17].

At the diachronic level, and over several decades examined through Annahar and Assafir, our results indicate that jobs such as doctors, engineers, scientists, and lawyers consistently favored males, while paid care occupations like teachers, nurses, secretaries, and artists, always preferred females. Those trends reflect that Lebanese society continues to adopt traditional views of the masculine (risk taking) versus the feminine (caring). However, there is an interesting shift in perceptions across time observed in the two newspapers, indicating changing perceptions with respect to female association to medical professions. Those results, which persist even after accounting for grammatical gender, can be corroborated by existing research indicating that in recent times, females are venturing into careers in the biological sciences as opposed to the mathematically based sciences because the latter careers were perceived to be less people-oriented and to have less value to society [9,10].

Even though our results validate the effectiveness of our adapted bias quantification methods in capturing the socio-linguistic patterns reflecting the pervasive nature of gender stereotypes, our work is not without limitations. For example, we have seen that that the corpora size can impact the WEAT test results, where we have noticed that the WEAT tests show statistical significance with an increased text size starting from the 1960s in the Annahar dataset. This finding is consistent with another study by [21], which shows that biases might be more reflected in word embedding models trained using bigger corpora.

Another major limitation of the bias quantification methods used in our research is the possible sensitivity of the bias results to the choice of the word lists and word pairs, where words might have multiple meanings based on the context they were used in the training data. Word embedding techniques face difficulties in handling polysemous words with various distinct meanings, such as the Arabic term "اب", which can denote either “August" or “father", contingent upon the context wherein it surfaces. When the training corpus contains instances where polysemous words manifest diverse meanings, the word embedding model conflates and combines the disparate semantic associations into a singular vector representation. To explain, the embedding vector for "اب" joins the semantics of both the August month and the familial relationship, which would impact the bias measurement where the vector possesses the concept of “father" and could contain associations stemming from the “August" word. Additionally, another limitation in the analysis of occupation bias could be the use of masculine forms to represent both female and male occupations. In formal contexts, masculine terms are often employed to address both genders. For example, the term "صيدلي" (male pharmacist) is commonly used to refer to individuals of both genders.

As an extension to this work, an interesting idea is to incorporate additional data sources to expand the scope of our study using social media data, literary works, and educational materials. This would be merited as our chosen datasets might not exhaustively reflect the wide variety of Arabic texts. Whilst the current paper provides a proof of concept that other researchers can benefit from and extend to more expansive corpora for further investigations, we believe that the sampling adopted in this work can help mitigate potential biases across several layers. For example, the chosen corpora provides a sampling across various cultures such as the Levantine region, North Africa,and the GCC region. The corpora also provides a sampling across time, with An-Nahar and As-Safir being historical archives whilst the online corpora being more recent. They also provide a sampling in terms of robustness of text, with Annahar and Assafir containing a prevalent pattern of OCR errors, whilst the online corpora presenting without any spelling errors. Finally, we provide a sampling across authorship, where newspapers are authored by professional journalists, whilst Wikipedia being open to contributors from the public. Moreover, we believe any potential biases were not significantly impactful because the interpretations we obtained align with existing studies around persistent gender biases especially across occupations ([2,17,20]).

Despite these limitations, our research fills a significant gap in the existing literature on bias analysis in Arabic word embeddings in two significant ways. We present the first comprehensive study tailoring bias quantification methods like Direct Bias and WEAT to the unique challenges posed by the Arabic language’s grammatically gendered nature, enabling reliable measurement of gender bias in Arabic text corpora. We also provide valuable insights into the nature and extent of gender biases reflected in Arabic text, an area largely unexplored in prior work focusing primarily on English and other languages. Despite that the historical archives presented with OCR errors, the interpretations revealed by our synchronic and diachronic variations across time and corpora are corroborated by existing results from the social sciences that align with the stereotypical gender-based norms affecting women’s careers. This opens avenues to extend the methodology presented in this manuscript to explore other biases or polarization in the Arab world that affect our perception of conflict, not least the polarization in how political and armed conflicts are perceived in Arabic discourse across time and regions.

## Conclusion

In this manuscript, we present an adaptation of the Direct Bias and WEAT bias quantification tests previously developed for the English language. By extending the chosen tests to word embeddings in Arabic, a low resource and grammatically gendered language, we focused on quantifying gender biases with respect to occupation. Using various datasets such as news archives, electronic newspapers, and the Arabic Wikipedia, our results reveal a consistent bias with respect to several occupations across most of the datasets. For example, occupations such as doctor, lawyer, representative/deputy, writer, and painter have been shown to consistently display a bias toward males. In contrast, jobs such as nurse, house cleaner, maid, secretary, and dancer consistently lean toward women. Our WEAT tests reveal associations across all datasets between career, science, and intellectual-related terms with men. Simultaneously, they reveal associations between family and art-related terms with women. To understand the impact of the grammatical component on bias computations, we applied the grammatical component of Arabic word embeddings and reproduced the bias quantification tests. The results suggest that, while grammatical gender impacts bias computations to some extent, the semantic gender biases remain substantially present even after disentanglement. In future work, we aim to expand our investigation by incorporating more diverse data sources, such as social media, archived literary works, and educational materials. Our initial exploration also calls for expanding the scope of bias quantification, to investigate the extent to which existing biases in word embeddings affect other NLP downstream tasks such as automatic translation and document ranking, to name a few.

## Supporting information

S1 Text (PDF)

## References

[1] Al-Dulaimi AH. Ultimate Arabic news dataset. Mendeley Data. 2022 Sept 21.

[2] Asi YM. Women at work in the Arab world: trends, gaps, and effects on the region. Arab Center Washington DC. 2022. Available from: https://arabcenterdc.org/resource/women-at-work-in-the-arab-world-trends-gaps-and-effects-on-the-region/

[3] Bolukbasi T, Chang KW, Zou JY, Saligrama V, Kalai AT. Man is to computer programmer as woman is to homemaker? debiasing word embeddings. Adv Neural Inf Process Syst. 2016;29.

[4] Boudchiche M, Mazroui A. Evaluation of the ambiguity caused by the absence of diacritical marks in Arabic texts: statistical study. In: 2015 5th International Conference on Information & Communication Technology and Accessibility (ICTA). 2015 Dec 21. IEEE; 2015. p. 1–6.

[5] Caliskan A, Bryson JJ, Narayanan A. Semantics derived automatically from language corpora contain human-like biases. Science 2017 Apr 14;356(6334):183–6. doi: 10.1126/science.aal4230 28408601

[6] Chennoufi A, Mazroui A. Morphological, syntactic and diacritics rules for automatic diacritization of Arabic sentences. J King Saud Univ-Comput Inf Sci. 2017 Apr 1;29(2):156–63.

[7] Dev S, Phillips J. Attenuating bias in word vectors. In: The 22nd International Conference on Artificial Intelligence and Statistics 2019 Apr 11. PMLR; 2019. p. 879–87.

[8] Doughman J, Abu Salem F, Elbassuoni S. Time-aware word embeddings for three Lebanese news archives. In: Proceedings of the Twelfth Language Resources and Evaluation Conference 2020 May. p. 4717–25.

[9] Eccles JS. Gender roles and women’s achievement-related decisions. Psychol Women Quart 1987 Jun;11(2):135–72. doi: 10.1111/j.1471-6402.1987.tb00781.x

[10] Eccles JS. Understanding women’s educational and occupational choices: applying the eccles et al. model of achievement-related choices. Psychol Women Quart. 1994 Dec;18(4):585–609.

[11] Fairclough N, Wodak. critical discourse analysis. The Routledge handbook of discourse analysis 2013 Jun 17. Routledge; 2013.

[12] Firth JR. A synopsis of linguistic theory 1930-1955. Studies in linguistic analysis, Special Volume/Blackwell; 1957.

[13] Font JE, Costa-Jussa MR. Equalizing gender biases in neural machine translation with word embeddings techniques. arXiv preprint 2019 Jan 10

[14] Garg N, Schiebinger L, Jurafsky D, Zou J. Word embeddings quantify 100 years of gender and ethnic stereotypes. Proc Natl Acad Sci U S A 2018 Apr 17;115(16):E3635–644. doi: 10.1073/pnas.1720347115 29615513 PMC5910851

[15] Gonen H, Goldberg Y. Lipstick on a pig: Debiasing methods cover up systematic gender biases in word embeddings but do not remove them. arXiv preprint 2019 Mar 9

[16] Greenwald AG, McGhee DE, Schwartz JL. Measuring individual differences in implicit cognition: the implicit association test. J Personal Soc Psychol 1998 Jun;74(6):1464–80. doi: 10.1037//0022-3514.74.6.1464 9654756

[17] Ibourk A, Elouaourti Z. Revitalizing women’s labor force participation in North Africa: an exploration of novel empowerment pathway. Int Econ J 2023 Jul 3;37(3):462–84. doi: 10.1080/10168737.2023.2227161

[18] Jbene M, Tigani S, Saadane R, Chehri A. 2021 International Conference on Decision Aid Sciences and Application (DASA) 2021 Dec 7. IEEE; 2121.

[19] Kay M, Matuszek C, Munson SA. Unequal representation and gender stereotypes in image search results for occupations. In: Proceedings of the 33rd Annual ACM Conference on Human Factors in Computing Systems 2015 Apr 18. p. 3819–28.

[20] Khafagy F, Abdel Khalik Z. Women’s economic justice and rights in the Arab Region. UN Women, 2021. Available from: https://arabstates.unwomen.org/sites/default/files/Field

[21] Lauscher A, Glavaš G. Are we consistently biased? multidimensional analysis of biases in distributional word vectors. arXiv preprint 2019 Apr 26

[22] Lauscher A, Takieddin R, Ponzetto SP, Glavaš G. AraWEAT: Multidimensional analysis of biases in Arabic word embeddings. arXiv preprint 2020 Nov 3

[23] McCurdy K, Serbetci O. Grammatical gender associations outweigh topical gender bias in crosslinguistic word embeddings. arXiv preprint 2020 May 18

[24] McEnery T, Hardie A. Corpus-based studies of synchronic and diachronic variation. Corpus Linguist: Method Theory Pract. 2011:94–121.

[25] Mikolov T, Chen K, Corrado G, Dean J. Efficient estimation of word representations in vector space. arXiv preprint 2013

[26] Mourad A. Gendered bias detection. GitHub repository, 2023. Available from: https://github.com/aiamourad/AraGenderBias

[27] Obeid O, Zalmout N, Khalifa S, Taji D, Oudah M, Alhafni B, et al. CAMeL tools: an open source python toolkit for Arabic natural language processing. In: Proceedings of the Twelfth Language Resources and Evaluation Conference 2020 May. p. 7022–32.

[28] Rudinger R, Naradowsky J, Leonard B, Van Durme B. Gender bias in coreference resolution. arXiv preprint 2018 Apr 25

[29] Omrani Sabbaghi S, Caliskan A. MMeasuring gender bias in word embeddings of gendered languages requires disentangling grammatical gender signals. In: Proceedings of the 2022 AAAI/ACM Conference on AI, Ethics, and Society 2022 Jul 26. p. 518–31.

[30] Wallace BC, Paul MJ. “Jerk” or “Judgemental”? Patient perceptions of male versus female physicians in online reviews association for the advancement of artificial intelligence (www.aaai.org). 2016.

[31] Wikipedia. Arabic Wikipedia Data. 2023. Available from: https://dumps.wikimedia.org/arwiki/

[32] Zenodo. Detecting gender bias in arabic text through word embeddings. 2024. Available from: doi: 10.5281/zenodo.1377247940163494

[33] Zhou P, Shi W, Zhao J, Huang KH, Chen M, Cotterell R, Chang KW. Examining gender bias in languages with grammatical gender. arXiv preprint 2019 Sep 5

