# Supplementary material for: Detecting gender bias in Arabic text through word embeddings
Source: PLoS One. 2025 Mar 31;20(3):e0319301. doi: 10.1371/journal.pone.0319301 (PMC11957338; doi:10.1371/journal.pone.0319301)
Supplement: S1 Text — (PDF) [file pone.0319301.s001.pdf]

Supplementary Materials

Corresponding code

To reproduce our experiments, readers can refer to the code publicly available at https://github.com/aiamourad/AraGenderBias

This repository includes:

- Bias quantification codes (Direct Bias and WEAT algorithms)
- Grammatical gender disentanglement code
- Plots scripts and notebooks

Corresponding datasets

1. Lebanese news archives (Annahar and Assafir) were obtained as OCR'ed text from images corresponding to microfilms from the American University of Beirut (AUB) Libraries. The collection and use of these datasets complied with the copyright and term of use instructions published here. Specifically, because the AUB is a third-party organization and legally restricts the sharing and copying of the actual data sets onto external public repositories, we have instead provided a minimal dataset consisting of the word embeddings trained on these archives. The word embeddings are publicly available in the following Zenodo repository: https://doi.org/10.5281/zenodo.13772479

2. Arabic Wikipedia: is publicly available at https://dumps.wikimedia.org/arwiki/. The use of Wikipedia data complies with the Creative Commons Attribution-ShareAlike 3.0 Unported License (CC BY-SA 3.0) and the GNU Free Documentation License (GFDL).

3. The Ultimate Arabic News Dataset: is publicly available at https://data.mendeley.com/datasets/jz56k5wxz7/2. The creators of this dataset have specified terms for research purposes.

4. The Moroccan News Articles Dataset (MNAD) is publicly available at https://github.com/J-Mourad/MNAD. Our use of the MNAD dataset adheres to the terms set by the dataset creators for academic and non-commercial research activities.

5. The raw values used to build Figures 2 till 10 are deposited in datasets in the GitHub directory found here: https://github.com/aiamourad/AraGenderBias/tree/main/paper_stats

6. The values behind all statistical measures reported in Table 5 can be generated dynamically using available code and data as follows. Table 5 presents WEAT statistics have been computed using word embedding models. The values appearing in this table can be reproduced by utilizing the same word embedding models found in https://doi.org/10.5281/zenodo.13772479 and executing the code provided in the

WEAT_Stimuli.ipynb file found here
https://github.com/aiamourad/AraGenderBias/blob/main/WEAT%20Stimuli.ipynb

7. Table 6 statistics are computed using the bias values provided in the GitHub link found here:
https://github.com/aiamourad/AraGenderBias/tree/main/paper_stats/Table%206. The bias values can be reproduced by executing the code provided in the Occupation Bias.ipynb file found here:
https://github.com/aiamourad/AraGenderBias/blob/main/Occupation%20Bias.ipynb. This code requires the embedding models provided earlier over
here: https://doi.org/10.5281/zenodo.13772479 and using the list of nouns provided
here: https://github.com/aiamourad/AraGenderBias/tree/main/nouns.
